# Cenani-Lenz syndactyly syndrome - a case report of a family with isolated syndactyly

**DOI:** 10.1186/s12881-018-0646-1

**Published:** 2018-07-24

**Authors:** Dineshani Hettiaracchchi, Carine Bonnard, S. M. A. Jayawardana, Alvin Yu Jin Ng, Sumanty Tohari, Byrappa Venkatesh, Bruno Reversade, Roshni Singaraja, V. H. W. Dissanayake

**Affiliations:** 1 Human Genetics Unit, Faculty of Medicine, University of Colombo, 25 Kinsey Place, Colombo, 08 Sri Lanka; 2 Institute of Medical Biology, A*STAR, Singapore, Singapore; 3 Faculty of Medicine and Allied Sciences, Rajarata University of Sri Lanka, Anuradhapura, Sri Lanka; 4 Institute of Molecular and Cell Biology, A*STAR, Singapore, Singapore

**Keywords:** Cenani-Lenz syndactyly syndrome, Whole exome sequencing, LRP4 gene

## Abstract

**Background:**

Cenani-Lenz Syndactyly (CLS) syndrome is a rare autosomal recessive disorder characterized by syndactyly and oligodactyly of fingers and toes, disorganization and fusion of metacarpals, metatarsals and phalanges, radioulnar synostosis and mesomelic shortness of the limbs, with lower limbs usually being much less affected than upper limbs.

**Case presentation:**

we report here two patients, born to consanguineous Sri Lankan parents, present with bilateral postaxial oligodactyly limited to upper limbs. While the proband has no noticeable facial dysmorphism, renal impairments or cognitive impairments, his affected sister displays a few mild facial dysmorphic features. Whole exome sequencing of the proband showed a novel deleterious homozygous mutation (c.1348A > G) in the *LRP4* gene, resulting in an Ile450-to-Val (I450V) substitution.

**Conclusion:**

This recessive mutation in *LRP4* confirmed the diagnosis of CLS syndrome in two patients present with isolated hand syndactyly. This is the first reported case of CLS syndrome in a family of Sri Lankan origin.

**Electronic supplementary material:**

The online version of this article (10.1186/s12881-018-0646-1) contains supplementary material, which is available to authorized users.

## Background

Cenani-Lenz Syndactyly syndrome (CLS, OMIM 212780) is a rare autosomal recessive disorder characterized by syndactyly and oligodactyly of fingers and toes, disorganization and fusion of metacarpals, metatarsals and phalanges, radioulnar synostosis and mesomelic shortness of the upper limbs, with lower limbs usually being much less severely affected than upper limbs [1–3]. It was first described in 1967 in two bothers with total syndactyly of the hands (‘spoon hands’), bilateral radioulnar synostosis and metacarpal fusion [1]. Facial involvement includes a prominent forehead, hypertelorism, downslanting palpebral fissures and micrognathia [4]. There have also been reports of renal hypoplasia or agenesis in over 50% of CLS families [5, 6]. The genetic etiology of CLS has been mapped to CLS1 locus on chromosome region 11p11.2–q13.1 and is thought to result from homozygous or compound heterozygous mutations in the *LRP4* gene [6, 7]. The encoded protein LRP4 (OMIM 604270) belongs to the family of low-density lipoprotein (LDL) receptors and acts as an antagonist to LRP5 and LRP6 in the Wnt signaling cascade, a function that is lost when the gene is mutated. Wnt signaling is known to be essential for embryonic limb patterning and kidney development [8, 9]. Here we report the first confirmed CLS family of Sri Lankan origin.

## Case presentation

**Fig. 1:**
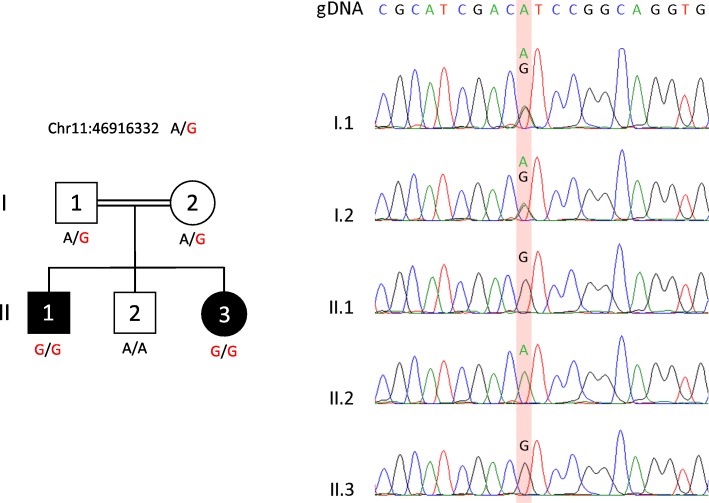
Pedigree of a consanguineous Sri Lankan family with two affected siblings with germline homozygous mutation in *LRP4* with sanger sequencing results

**Fig. 2:**
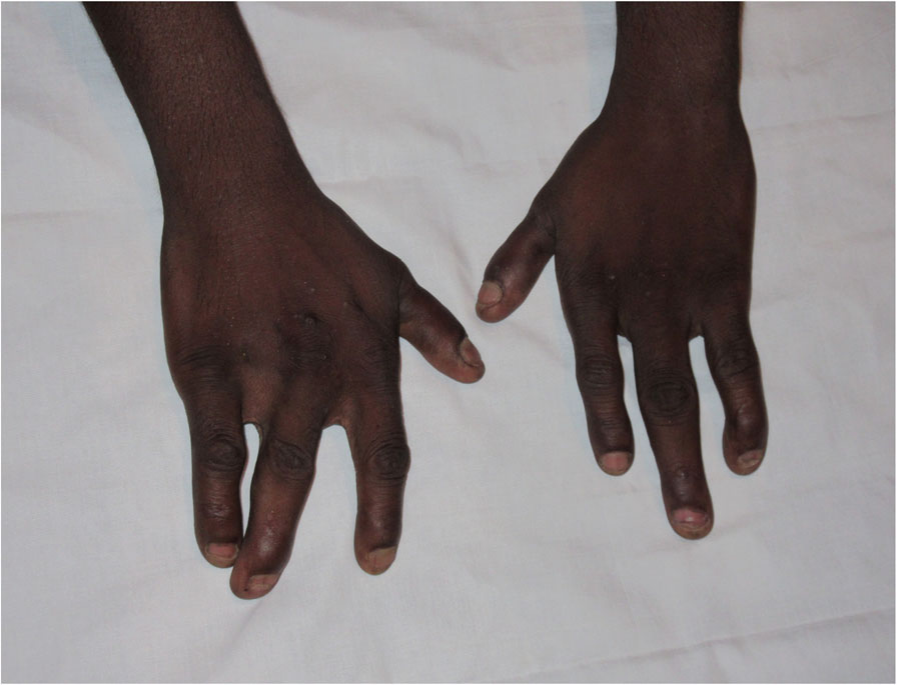
Hand photo of proband II.1 showing bilateral Syndactyly

**Fig. 3:**
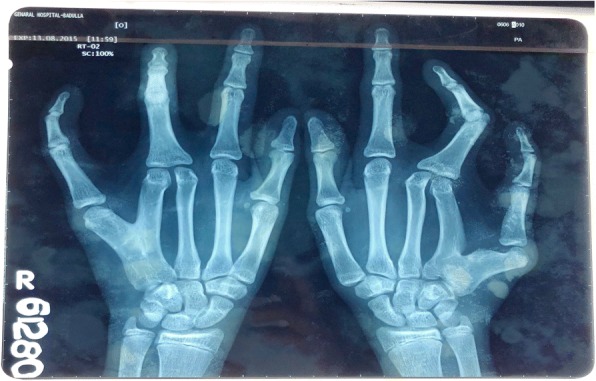
X- ray findings of the proband II.1 with fused 4th and 5th metatarsal bones

**Fig. 4:**
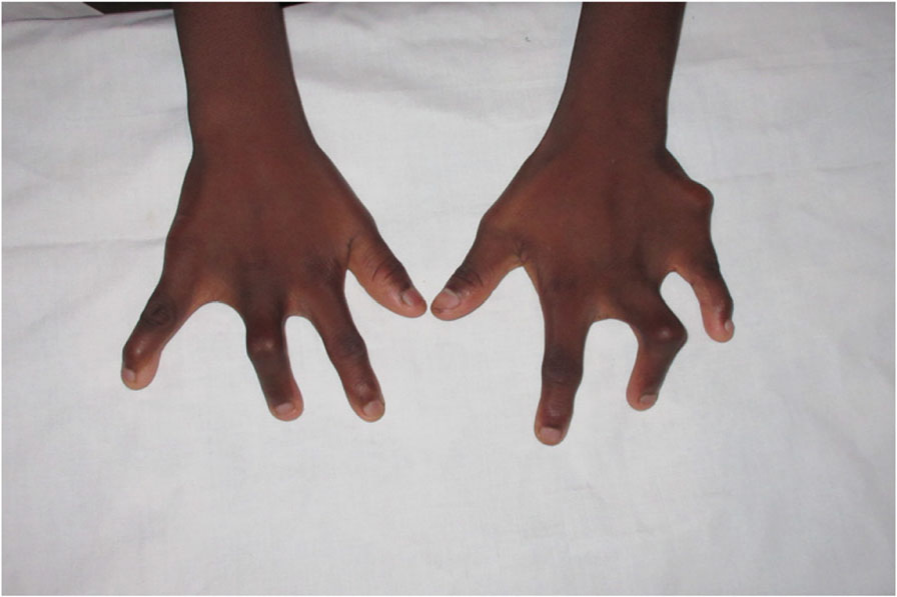
Hand photo of patient II.3 showing similar deformity as proband II.1

The proband (II.1), a 22-year-old male, was the eldest son of three children born to 1st degree consanguineous parents of Sri Lankan origin (Fig. 1). Pregnancy and delivery were uneventful. He was diagnosed to have bilateral postaxial oligodactyly limited to upper limbs at birth. Radiological studies showed bilateral fusion of the 4th and 5th metacarpal bones (Figs 2 and 3). He has no noticeable facial dysmorphism, renal impairments or cognitive impairments. The second child (II.2), a 16-year-old boy, was normal. The youngest child (II.3), a 13-year-old girl, also has postaxial oligodactyly (Fig. 4) and a few mild facial dysmorphic features. Both patients do not show visible lower limb deformities or oligodactyly. By whole exome sequencing of the proband, we identified a deleterious homozygous mutation in *LRP4* c.1348A > G, p.Ile450Val. Mutations in this gene were reported to cause CLS syndrome.

## Whole exome sequencing (WES) method and results

Following written informed consent from the proband and the family, genomic DNA was obtained from venous blood from each member. One microgram of genomic DNA per sample was used for exome capture with Agilent Technologies SureSelectXT All Human ExonV6 Kit. DNA was sheared to target an average fragment size of 200 bp and amplified as per SureSelect protocol. The exome library was prepared on an Ion OneTouch System and sequenced on an Ion Proton instrument (Life Technologies, Carlsbad, CA, USA) using one Ion PI chip.

Sequence reads were aligned to the human reference genome (Human GRCh37 (hg19) build) using Torrent Mapping Alignment Program (TMAP) from the Torrent Suite (v5.0.2). The variants were called using the Torrent Variant Caller (TVC) plugin (v5.0.2), and were annotated with the associated gene, location, quality-score, coverage, predicted functional consequences, protein position and amino acid changes, SIFT [10], PolyPhen2 [11], Grantham [12] and M-CAP [13] prediction scores, phyloP conservation scores [14] and 5000 genomes Minor Allele Frequencies. Variants were filtered for common SNPs using the NCBI’s “common and no known medical impacts” database (ftp://ftp.ncbi.nlm.nih.gov/pub/clinvar/vcf_GRCh37/) and the Exome Aggregation Consortium (ftp://ftp.broadinstitute.org/pub/ExAC_release/release0.2/). We next removed variants that were present in greater than 1% of the previously 534 sequenced samples.

For the proband analysis, 16.7 Gb were sequenced with an average read length of 177 bp. An average coverage of 196× was achieved over the exome with 97% of the bases covered at least 20 × .

A total of 949 heterozygous, compound heterozygous and homozygous variants were identified across protein-coding exons, UTRs, splice sites and flanking introns (Additional file 1: Table S1). Based on the fact that the two affected siblings, male and female, were born to consanguineous parents, we favored an autosomal recessive mode of inheritance. Only 44 homozygous variants remained, including 12 that were exonic. Based on the Genome Aggregation Database (gnomAD, http://gnomad.broadinstitute.org/), 4 out of the 12 exonic variants have not been identified in population, including a novel missense mutation in *LRP4* (c.1348A > G, p.Ile450Val). This variant was also not detected in our “in-house” database containing WES results of 534 patients who mainly originated from Middle-East. We predicted this variant to be pathogenic since its M-CAP score was 0.103, which is superior to the recommended pathogenicity threshold of 0.025 [13] (Additional file 1: Table S1). Moreover, the change of Isoleucine into Valine (p.Ile450Val) is expected to be deleterious since this amino-acid is highly conserved throughout evolution. Finally, mutations in *LRP4* were previously reported to cause Cenani-Lenz Syndactyly syndrome, supporting our finding. Using Sanger sequencing, this germline mutation was confirmed to segregate with the disease in all 5 family members (Fig. 1).

## Discussion and conclusion

Cenani-Lenz syndrome is a rare autosomal recessive disorder characterized by syndactyly and oligodactyly of fingers and toes. One gene underlying syndactyly is low-density lipoprotein receptor-related protein 4 (*LRP4*; OMIM 604270; RefSeq: NR_038909.1). Specifically, variants in the *LRP4* gene are responsible for Cenani-Lenz syndrome (CLS; OMIM 212780) and Sclerosteosis 2 (OMIM 614305). CLS is characterized by bilateral complex syndactyly of the hands (carpal, metacarpal and digital synostoses, disorganization of the carpal bones, digital reduction) and feet (toe syndactyly, metatarsal fusion, absent metatarsals), renal abnormalities, and dysmorphic facial features [1, 4, 15].

In this family two of the three siblings had shown isolated hand syndactyly with no lower limb or renal involvement. Thus far, variants in the *LRP4* gene have been reported only in one child with isolated hand syndactyly in literature. It has been recently suggested that variants in the fourth β-propeller of the extracellular protein domain may cause a phenotype distinct from previously characterized LRP4 variants, which has a more diverse phenotypic variation [16]. The genetic basis of several types of isolated Syndactyly has been identified but the genes underlying isolated syndactyly in many families remain to be discovered [17].

In conclusion we report a deleterious homozygous variant in *LRP4* c.1348A > G, p.Ile450Val. This variant may alter the ability of LRP4 to inhibit Wnt signaling, resulting in CLS type syndactyly. Hence our findings suggest that isolated syndactyly is a rare presentation of CLS.

## Additional file

Additional file 1: **Table S1.** Variant data – Data related to Whole Exome Sequencing (WES). (XLSX 274 kb)
